# The Counterproductive Effect of Right Anodal/Left Cathodal Transcranial Direct Current Stimulation Over the Dorsolateral Prefrontal Cortex on Impulsivity in Methamphetamine Addicts

**DOI:** 10.3389/fpsyt.2022.915440

**Published:** 2022-06-22

**Authors:** Xiaoyu Jiang, Yu Tian, Zhiling Zhang, Changwei Zhou, Jiajin Yuan

**Affiliations:** ^1^The Affect Cognition and Regulation Laboratory (ACRLab), Institute of Brain and Psychological Sciences, Sichuan Normal University, Chengdu, China; ^2^Psychological Correction Center, Sichuan Ziyang Drug Rehabilitation Center, Ziyang, China

**Keywords:** impulsivity, methamphetamine, transcranial direct current stimulation, dorsolateral prefrontal cortex, two-choice oddball

## Abstract

The current study aimed to evaluate the effect of transcranial direct current stimulation (tDCS) over the dorsolateral prefrontal cortex (DLPFC) on behavioral impulsivity in methamphetamine addicts. Forty-five methamphetamine addicts were recruited and randomly divided into active tDCS and sham tDCS groups to receive a daily tDCS intervention for 5 days, with the intensity set to 2 mA for the active group and 0 mA for the sham group. Anodal and cathodal electrodes were, respectively, placed over the right and left DLPFC. Behavioral impulsivity in methamphetamine addicts was examined by the 2-choice oddball task at 3-time points: before tDCS intervention (baseline), after the first intervention (day 1), and after 5 repeated interventions (day 5). Besides, twenty-four healthy male participants were recruited as the healthy controls who completed a 2-choice oddball task. Analysis of accuracy for the 2-choice oddball task showed that behavioral impulsivity was counterproductively increased in the active group, which was shown by the decreased accuracy for the deviant stimulus. The results suggested that the present protocol may not be optimal and other protocols should be considered for the intervention of methamphetamine addicts in the future.

## Introduction

Substance use disorders are prevalent health problems that are accompanied by mental disorders (1) and physical dysfunction (2), even underlying factors in criminal behavior (3). Individuals who chronically use methamphetamine exhibit higher behavioral impulsivity (4) which may result in constant drug use and relapse (5, 6). Behavioral impulsivity or behavioral disinhibition refers to the inability to inhibit a prepotent action (7). Previous studies have found that methamphetamine addicts exhibit higher behavioral impulsivity than healthy controls (8, 9), which persisted about 10 months after methamphetamine addicts abstained naturally (10). Behavioral inhibition is associated with most current therapies for methamphetamine addiction, which treat individuals by increasing their behavioral inhibition ability (11). Therefore, it is expected that a robust therapy outcome can be obtained by decreasing the behavioral impulsivity of methamphetamine addicts.

Methamphetamine addicts have shown structural (12, 13), metabolic (14), and functional (15) abnormalities in the frontal cortex, such as the dorsolateral prefrontal cortex (DLPFC). The DLPFC plays a primary role in the execution and inhibition of behavior, and its impairment decreased the ability to inhibit behavior (16). Notably, recent evidence suggested that using transcranial direct current stimulation (tDCS) to stimulate the DLPFC decreases behavioral impulsivity in individuals with attention deficit hyperactivity disorder (17), Gambling Disorder (18), and healthy individuals (19). However, it is unclear whether tDCS may effectively decrease behavioral impulsivity in methamphetamine addicts.

Transcranial direct current stimulation is a method of non-invasive brain stimulation, which has been used in the intervention of various psychiatric disorders (20) and the enhancement of cognitive function (21). The protocol of tDCS is crucial to the effectiveness of the technique (22). Previous studies have found a variety of tDCS protocols effective in decreasing craving in methamphetamine addicts (23–25), such as bilateral tDCS over the DLPFC (right anodal/left cathodal). This protocol has been shown to be effective in decreasing the symptoms of addiction, impulsivity in some substance addictions (e.g., tobacco and cocaine), or psychiatric disorders (18, 26, 27). In addition, multi-session of tDCS intervention has been found more effective than one session (28). Therefore, it can be expected that multi-session bilateral tDCS over the DLPFC (right anodal/left cathodal) can effectively decrease impulsivity in methamphetamine addicts.

Based on the evidence above, we hypothesized bilateral tDCS over the DLPFC (right anodal/left cathodal) may decrease behavioral impulsivity in methamphetamine addicts. To test this hypothesis, the current study used a 2-choice oddball task to examine behavioral impulsivity, as it has been shown to be effective in measuring behavioral impulsivity (29). The 2-choice oddball task requires participants to respond to two types of stimuli accurately and then quickly: one is standard and the other is deviant. The ratio of standard to deviant stimuli is 4 to 1, which means participants would be more habitual to respond to the standard stimulus; when a deviant stimulus presents, participants would inhibit their habitual response. Therefore, the accuracy and response time (RT) for deviant stimulus can be served as indicators of behavioral impulsivity (30).

## Materials and Methods

### Participants

According to *a priori* computation of the required sample size in the current design using G*Power statistical software (31), 36 individuals are necessary for 0.95 statistical power, and 45 individuals were used in the current study. The effect size was set to a threshold of medium (i.e., 0.25), according to previous meta-analysis reports regarding the effect of tDCS on drug addiction (28, 32), and the alpha was set to 0.05.

Forty-five individuals with methamphetamine addiction were recruited from Sichuan Ziyang Drug Rehabilitation Center, Sichuan Province, China. They were found by the police when they took drugs for the last time, and then they received unified management and treatment in the drug rehabilitation center, and have no chance to take drugs for 2 years. Inclusion criteria included meeting the criteria of the Diagnostic and Statistical Manual of Mental Disorders, 5th edition, never using drugs other than methamphetamine, and no acute physical or mental illness. Exclusion criteria included history of multiple drug use, current methamphetamine use or medication, history of acute physical and mental problems (e.g., epilepsy, stroke, cardiovascular disease), presence of metal implants (e.g., electrodes, pacemakers, heart bypass), and history of brain stimulation interventions. Each methamphetamine addict was randomly assigned to an active tDCS group (*n* = 23) with a 2 mA current intensity or a sham tDCS group (*n* = 22) with a 0 mA current intensity, according to a computer-generated randomization sequence. The overall mean age of the methamphetamine addicts was 24.1 (SD = 2.13) years, 24.3 (SD = 1.57) years in the active group, and 24 (SD = 2.62) years in the sham group. Additionally, 24 healthy male participants were recruited as healthy controls. Their mean age was 25.2 (SD = 4.14) years. The three groups were matched in age, *F*_(2,66)_ = 1.129, *p* = 0.33, η*_*p*_*^2^ = 0.033.

**FIGURE 1:**
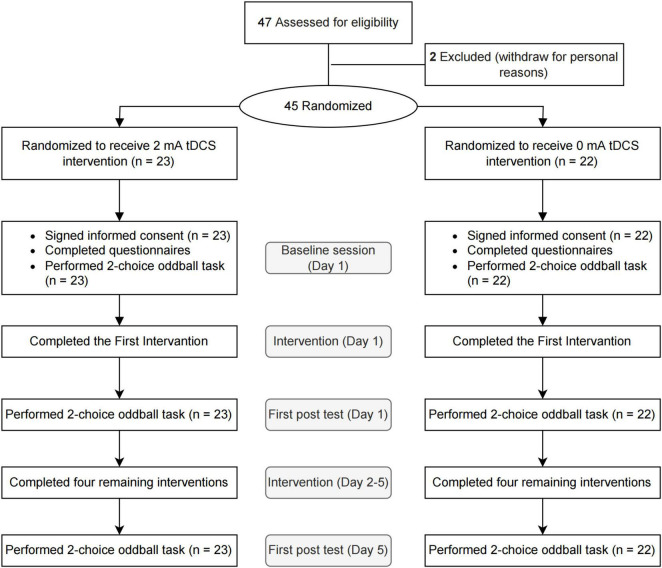
Flow-chart of the experimental procedure.

All participants were right-handed and had normal or corrected-to-normal vision. They voluntarily participated in the study and signed written informed consent before receiving the intervention. The current study has been registered on the platform of the China Trial Registration Center (Registration number: ChiCTR2100046112) and has been approved by the Ethical Committee of the Institute of Brain and Psychological Sciences, Sichuan Normal University in China. The experimental procedure was in line with principles of the Declaration of Helsinki and followed the Consolidated Standards of Reporting Trials (CONSORT) reporting guidelines; see Figure 1.

### Transcranial Direct Current Stimulation Procedure

“Direct currents of 2 mA generated by an electrical stimulator (Brain Premier tDCS Device; China) were applied through a pair of saline-soaked 1.5” round sponge electrodes for 20 min. In both active and sham groups, anodal and cathodal electrodes were placed over the right and left DLPFC, respectively (F4-F3), which was determined *via* the standard tDCS navigation system provided by the NeuStim NSS18 equipment of Neuracle Company (Changzhou, China). For the sham group, the direct current intensity is set to 0 mA and the intervention time is the same as the active group. To test the effectiveness of the sham protocol, after the intervention, participants were randomly selected and asked orally about their feelings. The tDCS intervention was performed for 5 sessions over 5 consecutive days. The experimenter who applied tDCS was blind to the study hypothesis but not to the setting of two groups (active vs. sham).

### Behavioral Measurement

The two-choice oddball task contained 200 trials, including 160 standard stimuli (“W”) and 40 deviation stimuli (“M”). Each trial started with a jittered fixation cross appearing at the center of the screen and varying from 500 to 1,500 ms. For the participants in each group, if the standard stimulus was presented, they were to press the “F” key with their left index finger as quickly as possible. If the stimulus was deviant, they were to press the “J” key with their right index finger. Before the task started, each participant completed 15 practice trials to familiarize themself with the procedure. To avoid the practice effect, the formal experiment did not start until participants achieved 100% accuracy in both standard and deviant stimuli during practice. At the end of the experiment, the accuracy was given as feedback to participants.

Behavioral impulsivity was primarily indicated by the accuracy of the deviant stimulus. In the methamphetamine addicts group, behavioral impulsivity was examined at 3-time points: before tDCS intervention (baseline), after the first intervention (day 1), and after 5 repeated interventions (day 5). Besides, healthy controls completed a 2-choice oddball task as the baseline impulsivity level of healthy individuals.

### Data Analysis

To verify the effectiveness of the manipulation, we collected the RT and accuracy from the 2-choice oddball task and used the baseline data to conduct a 2 × 2 mixed-design ANOVA, with stimulus (standard, deviant) as within-subject factor and group (healthy controls, methamphetamine addicts) as between-subject factor. To analyze the effect of tDCS on behavioral impulsivity in methamphetamine addicts, a 2 × 3 × 2 mixed-design ANOVA was used, with group (sham, active) as between-subject variable, session (baseline, day 1, day 5), and stimulus (standard, deviant) as within-subject variables. Potential group differences in demographic data and questionnaires were analyzed using independent samples *t*-tests, ANOVA, and Kruskal–Wallis tests, as appropriate.

According to the Greenhouse–Geisser method, the degrees of freedom for *F*-ratios that violate the spherical assumption are corrected. The false discovery rate (FDR) correction was used for *post-hoc* comparisons if statistically significant main or interaction effects appeared. All statistical analyses were performed in R (33). A 2-sided *p* < 0.05 was considered statistically significant, and the effect size was reported as partial η^2^ (η*_*p*_*^2^).

## Results

The mean abstinence duration was 153 (SD = 78.3) days in the active group, and 123 (SD = 58.6) in the sham group, and the 2 groups were overall matched in the duration of abstinence (*p* = 0.15). The three groups were matched on other demographic variables, demographic data are presented in Table 1.

**TABLE 1:** Demographic data of methamphetamine addicts undergoing active or sham tDCS and healthy controls.

	Methamphetamine addicts				
Characteristic	tDCS	Sham	Healthy controls	*F*/χ^2^	*p*-value
Sex	Male	Male	Male	NA	NA
Participants, No.	23	22	24	NA	NA
Age, mean (SD), y	24.3 (1.57)	24.0 (2.61)	25.3 (4.14)	1.19	1.31
Education^a^	2.30 (0.82)	1.91 (0.81)	2.38 (0.77)	4.85	0.09
Smoking	2.26 (1.10)	2.41 (1.05)	2.00 (1.06)	0.87	0.43
SAS	29.8 (8.24)	32 (6.87)	31.7 (3.71)	0.88	0.42
SDS	34.0 (5.59)	34.7 (7.17)	35.6 (7.23)	0.4	0.67
BIS	71.4 (14.9)	75.9 (18.6)	70.8 (12.1)	−0.81	0.42
Rehabilitation	153 (78.3)	123 (58.6)	NA	−1.47	0.15
Addiction^b^	2.56 (0.73)	2.5 (0.80)	NA	0.06	0.81

*^a^Unit for Education: denoted as 1 for primary school, educated for 6 years; 2 for junior high school, educated for 9 years; 3 for senior high school, educated for 12 years; 4 for college, educated for 16 years.*

*^b^Unit for Addiction: denoted as 1 for addicted for 2 years and below; 2 for addicted for 3–5 years; 3 for addicted for 6–10 years; 4 for addicted for 11 years and above. NA means not available.*

### Manipulation Check

**FIGURE 2:**
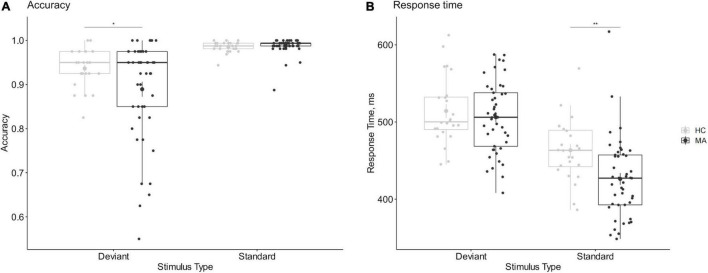
Comparison of behavioral impulsivity between methamphetamine addicts and healthy controls. **(A)** The accuracy of 2-choice oddball task. **(B)** The response time of 2-choice oddball task. **(A)** The accuracy of 2-hoice oddball. **(B)** The response time of 2-choice oddball. MA refers to methamphetamine addicts, HC refers to healthy controls. *Refers to 2-sided *p* < 0.05. **Refers to 2-sided p < 0.01. Error bars show the standard error of the mean. Enlarged dots refer to means.

Baseline data were used to check the effectiveness of manipulation. The mixed-design ANOVA of accuracy and RT in the 2-choice oddball task showed statistically significant group-by-stimulus interaction effects, accuracy, *F*_(1,67)_ = 4.204, *p* = 0.044, η*_*p*_*^2^ = 0.059; RT, *F*_(1,67)_ = 12.016, *p* < 0.001, η*_*p*_*^2^ = 0.152. The deviant stimulus had lower accuracy and longer RT relative to the standard stimulus in both samples (*p*s < 0.001), indicating that the experimental manipulation was effective and that the 2-choice oddball task could successfully measure behavioral impulsivity. Notably, although methamphetamine addicts and healthy controls showed similar accuracy in the standard stimulus (*p* = 0.606) and RT for the deviant stimulus (*p* = 0.479), the accuracy of the deviant stimulus (*p* = 0.033) and the RT for the standard stimulus (*p* = 0.003) was significantly lower in methamphetamine addicts compared with healthy controls, indicating that methamphetamine addicts showed higher behavioral impulsivity relative to healthy controls; see Figure 2.

### The Effect of Transcranial Direct Current Stimulation on 2-Choice Oddball Task

**FIGURE 3:**
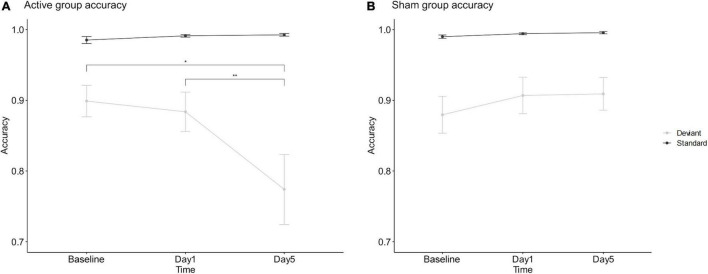
Mean accuracy of active tDCS group **(A)** and sham group **(B)**. *Refers to 2-sided *p* < 0.05. **Refers to 2-sided p < 0.01. Error bars show the standard error of the mean.

The mixed-design ANOVA of accuracy showed a statistically significant 3-way interaction among stimulus, session and group, *F*_(1.62,69.51)_ = 5.96, *p* = 0.007, η*_*p*_*^2^ = 0.122. The simple effects analysis found a significantly decreasing deviant stimulus accuracy (*p* < 0.035) and no significantly different standard stimulus (*p* > 0.296) after 5 days of interventions in the active group. Importantly, this significant stimulus-by-time interaction only found in the active group, *F*_(1.46,32.13)_ = 6.354, *p* = 0.009, η*_*p*_*^2^ = 0.224, but not in the sham group, *F*_(2,42)_ = 0.686, *p* = 0.509, η*_*p*_*^2^ = 0.032. These results indicated a significantly increased behavioral impulsivity after 5 consecutive days of interventions in the active group, and no difference in the sham group; see Figure 3.

The analysis of RT found no statistically significant three-way interaction among stimulus, time and group, *F*_(2,86)_ = 2.826, *p* = 0.065, η*_*p*_*^2^ = 0.062, except for a significant stimulus by time interaction, *F*_(2,86)_ = 21.876, *p* < 0.001, η*_*p*_*^2^ = 0.337, and a significant group by stimulus interaction, *F*_(1,43)_ = 6.105, *p* = 0.018, η*_*p*_*^2^ = 0.124. Further analysis revealed that, regardless of the active group or the sham group, the RT for the standard stimulus significantly decreased over time (both: *p*s < 0.006), while the RT for the deviant stimulus was not significantly different before and after the interventions (both: *p*s > 0.124).

## Discussion

The current study aimed to evaluate the effect of bilateral tDCS (right anodal/left cathodal) over DLPFC in decreasing behavioral impulsivity in methamphetamine addicts. Inconsistent with our hypothesis, the results suggested that the current protocol of bilateral tDCS counterproductively increased impulsivity in methamphetamine addicts. Specifically, after 5 consecutive days of intervention, the accuracy for deviant stimulus was significantly decreased in the active group, while the sham group did not.

Mounting evidence indicated that the DLPFC is closely linked to behavioral control. Previous studies have found that individuals with impaired DLPFC generally performed worse on executive measures relative to healthy individuals and individuals with damage in other brain regions (34). Moreover, some studies have found that stimulating the DLPFC of individuals through tDCS decreased the impulsivity of healthy (19), ADHD individuals (35), and gambling addicts (18), and suggested that using an appropriate tDCS protocol to stimulate DLPFC may effectively improve individuals’ impulse control or behavioral inhibition ability. Based on these findings, the current study selected the DLPFC as a tDCS target and expected this protocol to significantly decrease behavioral impulsivity in methamphetamine addicts. However, counterproductive results were observed, with a significant increase in behavioral impulsivity of methamphetamine addicts. These results indicated that using tDCS to stimulate the DLPFC does an effective method to modulate behavioral impulsivity, but the protocol that used bilateral tDCS (right anodal/left cathodal) over DLPFC may lead to up-modulation.

Methamphetamine addicts have severely impaired DLPFC relative to healthy individuals (36). Specifically, methamphetamine addicts had significantly lower gray matter thickness in the DLPFC region (12, 37, 38) and lower activation during behavioral inhibition tasks (39). One recent study observed that bilateral tDCS (right anodal/left cathodal) over DLPFC increased the activation of executive control networks in the resting state of methamphetamine addicts and decrease the craving of methamphetamine addicts (25). However, given the extent of damage to the DLPFC in methamphetamine addicts, it is possible that activating this region may overdraw their DLPFC activity and subsequently decrease their impulse control. For example, a warm-up usually improves performance in healthy people, but the same warm-up may deplete sick person and his/her subsequent performance. This may be one potential reason why a similar protocol decreased impulsivity in healthy individuals (40) and individuals with other psychological disorders (18) but led to counterproductive results in methamphetamine addicts.

Several limitations should be addressed in future work. First, although the current study used a 2-choice oddball task to assess impulsivity, no functional neuroimaging with tDCS intervention was collected, which led us unable to examine possible functional changes in the DLPFC. Therefore, future research can examine the current findings using neuroimaging techniques. Second, the current study measured baseline behavioral impulsivity in the HC group but they did not undergo tDCS intervention, leaving it not possible to examine how tDCS affects behavioral impulsivity in healthy people. In addition, the current study employed simple letters as stimuli (i.e., M and W) to ensure experimental control. However, previous studies have found that methamphetamine addicts have higher impulsivity to meth-related information (41, 42), so future work should consider selecting drug-related images as stimuli to improve the ecological validity of the study. Furthermore, the current study used only one active tDCS protocol (right anodal/left cathodal), which prevented us from exploring the effects of unilateral stimulation of the DLPFC on impulsivity in methamphetamine addicts. However, because the anodes and cathodes of tDCS may be associated with opposing neural effects (20) and the anodal tDCS may have different effects on the left DLPFC and the right DLPFC in methamphetamine addicts (22), additional tDCS protocols are needed in future studies to further investigate the lateralizing effects of tDCS on DLPFC function.

## Conclusion

The current study evaluated the effect of bilateral tDCS (right anodal/left cathodal) over the DLPFC on behavioral impulsivity in methamphetamine addicts and found a counterproductively increased impulsivity after the 5-day intervention in methamphetamine addicts. The results suggested that using tDCS to stimulate the DLPFC is an effective method to modulate behavioral impulsivity, but as it is counterproductive, the current protocol may not be optimal and other protocols should be considered for the intervention of methamphetamine addicts in the future.

## Data Availability Statement

The original contributions presented in this study are included in the article/Supplementary Material, further inquiries can be directed to the corresponding author.

## Ethics Statement

The studies involving human participants were reviewed and approved by the Ethical Committee of the Institute of Brain and Psychology Science, Sichuan Normal University. The patients/participants provided their written informed consent to participate in this study.

## Author Contributions

XJ and JY conceived and designed the current study. XJ and CZ collected the data and statistical analysis. XJ, YT, and JY interpreted the data. XJ, YT, ZZ, and JY wrote the final manuscript. All authors contributed to reviewed and approved the final manuscript.

## Conflict of Interest

The authors declare that the research was conducted in the absence of any commercial or financial relationships that could be construed as a potential conflict of interest.

## Publisher’s Note

All claims expressed in this article are solely those of the authors and do not necessarily represent those of their affiliated organizations, or those of the publisher, the editors and the reviewers. Any product that may be evaluated in this article, or claim that may be made by its manufacturer, is not guaranteed or endorsed by the publisher.
